# Genome ARTIST_v2—An Autonomous Bioinformatics Tool for Annotation of Natural Transposons in Sequenced Genomes

**DOI:** 10.3390/ijms232012686

**Published:** 2022-10-21

**Authors:** Alexandru Al. Ecovoiu, Alexandru Marian Bologa, David Ioan Mihail Chifiriuc, Andrei Mihai Ciuca, Nicoleta Denisa Constantin, Iulian Constantin Ghionoiu, Iulian Cristian Ghita, Attila Cristian Ratiu

**Affiliations:** 1 Department of Genetics, Faculty of Biology, University of Bucharest, 060101 Bucharest, Romania; 2 Independent Researcher, 060101 Bucharest, Romania; 3 Exenne Technologies SRL, 031215 Bucharest, Romania; 4 Accelerate Systems, London N1 7SR, UK

**Keywords:** *Drosophila melanogaster*, Genome ARTIST, natural transposons, insertion mapping, genome sequencing, bioinformatics

## Abstract

The annotation of transposable elements (transposons) is a very dynamic field of genomics and various tools assigned to support this bioinformatics endeavor have been developed and described. Genome ARTIST v1.19 (GA_v1.19) software was conceived for mapping artificial transposons mobilized during insertional mutagenesis projects, but the new functions of GA_v2 qualify it as a tool for the mapping and annotation of natural transposons (NTs) in long reads, contigs and assembled genomes. The tabular export of mapping and annotation data for high-throughput data analysis, the generation of a list of flanking sequences around the coordinates of insertion or around the target site duplications and the computing of a consensus sequence for the flanking sequences are all key assets of GA_v2. Additionally, we developed a set of scripts that enable the user to annotate NTs, to harness annotations offered by FlyBase for *Drosophila melanogaster* genome, to convert sequence files from .fasta to .raw, and to extract junction query sequences essential for NTs mapping. Herein, we present the applicability of GA_v2 for a preliminary annotation of P-element and hobo class II NTs and copia retrotransposon in the genome of *D. melanogaster* strain Horezu_LaPeri (Horezu), Romania, which was sequenced with Nanopore technology in our laboratory. We used contigs assembled with Flye tool and a Q10 quality filter of the reads. Our results suggest that GA_v2 is a reliable autonomous tool able to perform mapping and annotation of NTs in genomes sequenced by long sequencing technology. GA_v2 is open-source software compatible with Linux, Mac OS and Windows and is available at GitHub repository and dedicated website.

## 1. Introduction

Transposable elements (transposons) are mobile genetic elements that are found in almost all genomes of species and contribute to the evolution of genomes through genomic reshuffling, structural chromosome changes and implicit chromosome size modifications. In addition to these, transposons influence gene expression processes, causing species to respond differently to various environmental factors, sometimes even leading to phenotypic changes [1]. Through their insertional mutagenic potential, transposons can affect gene expression or even disrupt the coding sequences of genes. Unlike class I of natural transposons (NTs), those from class II can undergo spontaneous excisions inducing unstable mutations. Considering all the above, determining the precise genomic location of transposons insertions is a common experimental goal. To investigate the biology of NTs, the main strategy is to sequence a eukaryotic genome and to detect, through bioinformatics methods, canonical and new insertions. 

Even if the mapping of transposon insertions remains a challenging topic in bioinformatics because of the repetitive nature of these sequences, it is an active research area, with a large number of tools published in recent years [2]. Nowadays, massive efforts are made to sequence the genomes of many different organisms that are subsequently stored in dedicated databases, hence, providing huge amounts of data to be analyzed. Advances in NTs annotation projects still require new and improved bioinformatics approaches in order to develop more specific and precise tools for best suiting the annotators’ needs. Over the last several years, the bioinformatics tools that are able to detect and map NTs insertions were developed to operate primarily on high-throughput sequencing data [3,4]. Initially designed for mapping artificial transposon insertions, Genome ARTIST v1.19 (GA_v1.19) differs from most tools dedicated to transposable elements analysis due to its intuitive and clear graphical interface, as well as its high mapping efficiency and accuracy [5]. 

Considering the present scientific context, we improved GA_v1.19 in order to make it able to cope with the new trends in NTs research, involving high-throughput approaches and automating data exploration. Here, we present GA_v2, a new version that contains several additional features, which proved to be very effective when addressing high-throughput NTs mapping projects. The new implemented functions allow users to retrieve subsequences from specific references coordinates without a prior alignment with a query sequence; to extract a list of target site duplications (TSDs) or of flanking sequences consecutive to the alignment of a set of transposon-genome junction query (JQ) sequences versus reference sequences; to visualize the terminal genomic nucleotide (TGN) of insertion highlighted in green; to export a data table that includes the alignment score, insertions coordinates, hit genes, upstream and downstream genes and the TSD sequence generated by each insertion; to crop JQs around the site of insertion of NTs, which are essential for mapping; to convert .fasta files in .raw files; to annotate genes or NTs; and to convert reference sequences and annotations of the canonical genome of *Drosophila melanogaster* available in FlyBase [6] in a compatible format. On the other hand, GA_v2 run on x64 on Linux, Mac OS and Windows.

## 2. Results and Discussions

### 2.1. The New Features of GA_v2

#### 2.1.1. GA_v2 Upgrades Relative to GA_v1.19

The upgrades of GA_v2 comparative to the previous version are presented in Table 1. These permit an improved aligning and mapping experience, noticeable mainly when large genomes, such as the mammalian ones, are used as reference sequences and allow the user to access and explore the results in detail.

#### 2.1.2. GA_v2 Upgrades Relative to the Export of Annotation Data and Sequence Manipulations

All of the annotations associated with the mapping results and depicted in the graphical interface are now exportable in a tabular format, which permits various subsequent manipulations of the mapping/aligning data. In addition to the insertion coordinates and chromosomal localization, GA_v2 also exports the name of the associated transposon loaded to Transposon files, the hit gene and the upstream/downstream genes in the vicinity of the insertion, the alignment score and TSD nucleotide sequences of each NT insertion. By loading GA_v2 with a reference genome annotated with both genes and NTs, an insertion may be associated with other NTs or with any other annotated genomic features.

GA_v2 is able to retrieve flanking sequences of customizable lengths that are exported as .fasta files, so they may be readily used for various subsequent analyses, such as multiple alignments and consensus motifs detection. For this purpose, the user is expected to press the Export to FASTA button.

**Figure 1:**
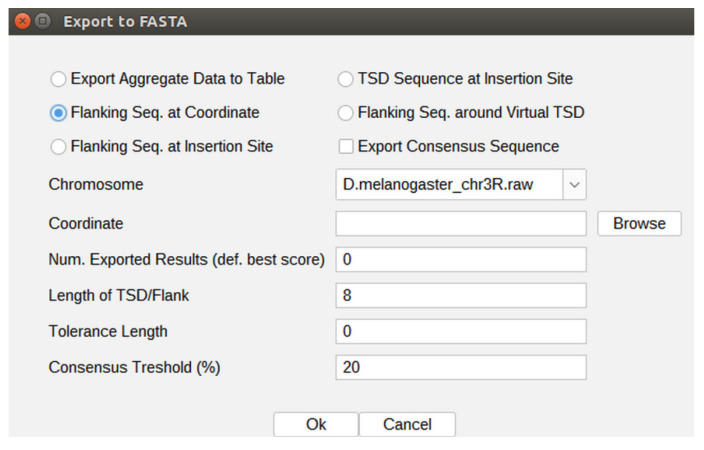
The Flanking seq. at the Coordinate function that enables the export of specified length nucleotide sequences centered on coordinates provided either directly or by uploading a predefined list through the Browse option.

Flanking sequences can be obtained around border nucleotides identified by the algorithm or around user-specified coordinates. For the latter, the Flanking Seq. at Coordinate option must be selected and then a chromosome must also be chosen from the drop-down list (Figure 1). A single coordinate can be specified in the adequate field or a .txt file can be loaded, where each line contains a coordinate.

A value for the length of the flanking sequence can be set in the Length of TSD/Flank field, where the default value of this parameter is 8, standing for the TSD length generated by insertion of the P-element in *D. melanogaster* genome. For example, if one changes this value to 20, then two local subsequences of 20 nucleotides centered on each coordinate of interest are considered for constituting the output sequences (one of the subsequences contains the respective query coordinate). Therefore, a list of specific contiguous sequences of 40 nucleotides will be extracted and exported as a .fasta file (the output file) for each of the nucleotide coordinates present in the input file.

If the exported flanking sequences are relatively short (a few tens of nucleotides) and are to be further aligned with GA_v2 for various purposes, such as local homology and oligonucleotide specificity, we recommend setting the value of picking depth parameter at a higher value than the default one, which is 1. Therefore, identical alignments standing for different coordinates in the reference genome may be reported in the list of results.

Similarly, consecutive to a post-alignment analysis, either a list of TSD nucleotide sequences or a list of TSDs encompassed by flanking sequences of preset lengths may be exported as a .fasta file. In this case, the input contains a list of JQs instead of chromosomal coordinates, each containing at least a transposon-genome or a transposon–transposon border. As mentioned above, the length of a TSD is customizable. 

**Figure 2:**
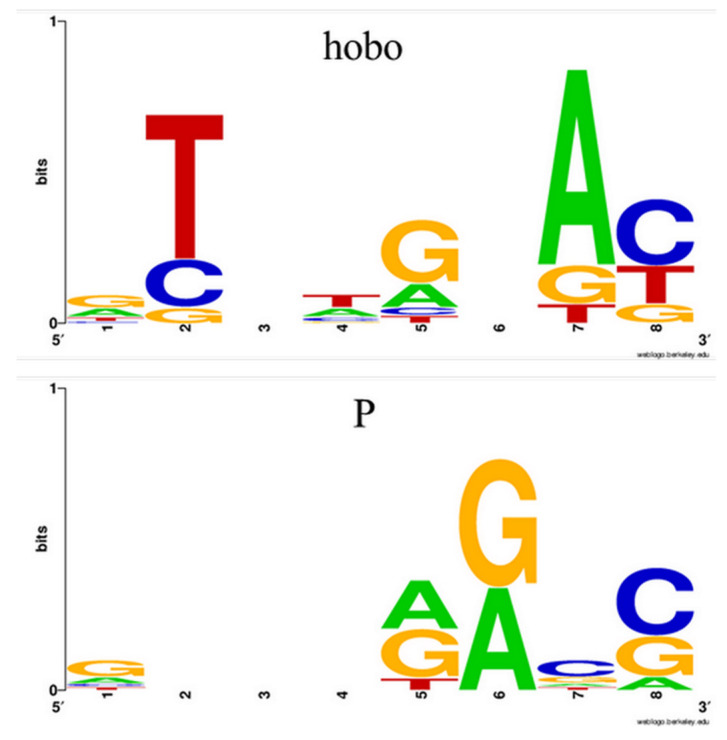
The sequence logos generated by WebLogo using the .fasta file containing sets of hobo (upper logo) and P-element (lower logo) transposons’ TSD sequences from the Horezu local *D. melanogaster* strain.

Using the TSD Sequence at Insertion Site function on data collected from the genome of Horezu_LaPeri (Horezu) local *D. melanogaster* strain from Horezu, Romania (BioProject/NCBI, accession PRJNA629549), we generated two lists of 13 hobos and, respectively, 9 P-element 8 bp TSD sequences. These lists were further loaded in WebLogo [7] and corresponding logos were computed (Figure 2). Regarding the hobo’s duplicated sequence, we obtained a consensus in which the second (T), the fourth (T), the seventh (A) and the last (C) positions are highly conserved, in accordance to the ones reported in the literature [8,9]. Relative to P-element, the TSD consensus is similar to those previously computed [10,11].

Starting from a list of TSDs or flanking sequences, a consensus sequence can be generated by GA_v2 using IUPAC nucleotide ambiguity code. The user must load a list of junction sequences in GA_v2, then activate TSD Sequence at Insertion Site, check Export Consensus Sequence and press the Ok button. The consensus sequence is exported in FASTA format and then may be used to scan against specific databases to find related consensus/motifs counterparts.

The consensus threshold value is set by default at 20% and is customizable. This value means that a specific nucleotide should be present in the same position in at least 20% of the exported sequences in order to be considered when selecting an IUPAC symbol for the consensus sequence. Obviously, if the value is set at 100%, the TSDs or the flanking sequences must be identical in order to obtain a consensus sequence. However, in this case, the consensus sequence would rather be a strict repetitive motif sequence, as it is the case of an invariable restriction site.

#### 2.1.3. GA_v2 Upgrades for Data Visualization

In GA_v2, the TGN at the junction border is highlighted in green, helping the user to identify the proper coordinate of insertion, mainly when the iPCR-derived sequence has transposon–genome–transposon structures.

GA_v2 automatically saves mapping/aligning results in the resources/outputs/ folder with the date and a name chosen by the user, not with aleatory names as in GA_v1.19. The results may be reviewed even with an empty GA_v2 package (containing no reference sequences). In addition, multiple results may be selected to be opened in the graphical interface.

GA_v2 package contains new scripts, which are available in the scripts directory. In contrast to GA_v1.19, the FlyBase_annotation.sh script that is found in the folder FlyBase allows the user to simultaneously associate multiple sets of annotations with the reference sequence of *D. melanogaster* genome. It uses .fasta.gz files downloaded from FlyBase that contain annotations of *D. melanogaster* genome such as genes, transposons, pseudogenes, predicted features, 3’UTRs and several others. The script simply needs to be run in the folder where are located the .fasta.gz files that contain the annotations and it will generate a chr_gene.fasta file, corresponding to each chromosomal arm. Generated .fasta files are loaded in GA_v2 together with the .raw files (previously downloaded from FlyBase FTP server). Therefore, the location of a transposon insertion may be interpreted in a more complex context of the genomic local landscape revealed by graphical interface of GA_v2. 

**Figure 3:**
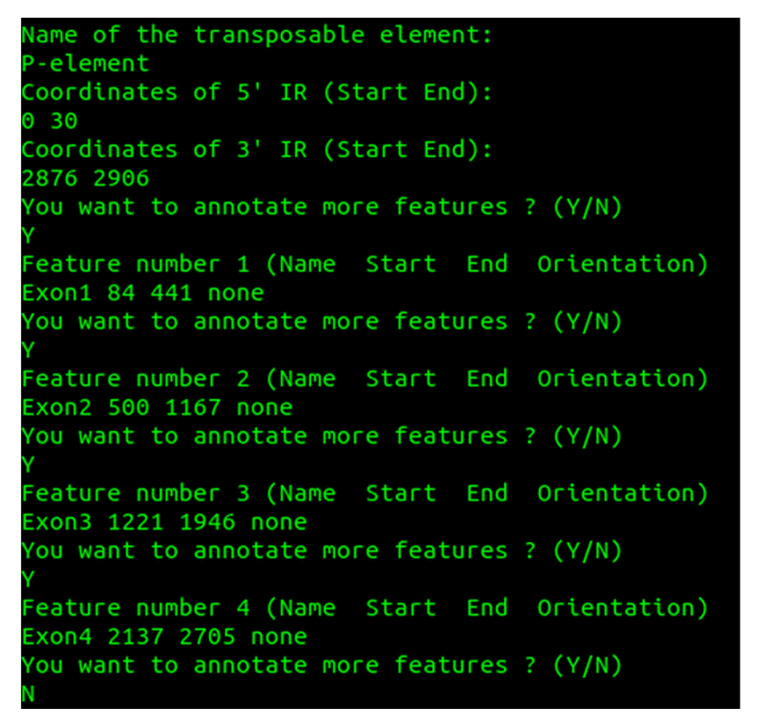
The *bash* terminal window showing the effect of TE_annotator.sh script when annotating the TIRs (5’ IR and 3’ IR) and exon regions of P-element.

**Figure 4:**
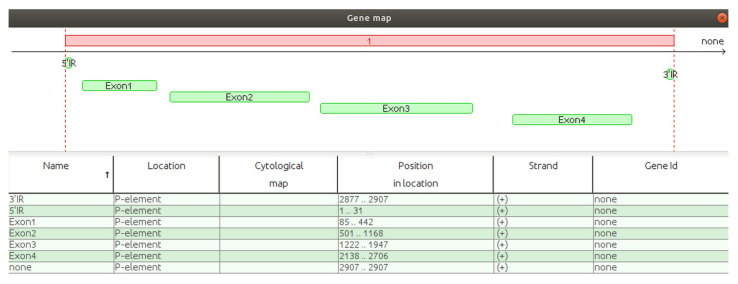
GA_v2 visualization of the annotation features implemented for P-element.

The script TE_annotator.sh automatizes manual annotations of transposons, allowing the user to define the terminal inverted repeats (TIRs) of NTs, genes and other annotations within the mobile element. The script must be run from “root folder”/resources/gene/ directly in the Linux terminal. The name of the transposon indicated in the appropriate terminal window should coincide with the name used in GA_v2 (we assume that one or more transposons have been preloaded in GA_v2 database). Before allowing the user to annotate the desired features, the script requires the coordinate intervals/ranges of the 5’ and 3’ ends to be specified. Figure 3 shows the terminal window into which the annotations of the natural transposon P-element were implemented using the TE_annotator.sh script, while in Figure 4 the graphical implementation of these annotations is depicted.

As previously stated, the annotation line referring to the first nucleotide of the sequence must start with the zero coordinate [5]. Note that when starting from zero, each coordinate must be subtracted by one position.

All the annotations implemented by these scripts are graphically depicted in green when they are overlapping with the query sequences. 

### 2.2. Case Study

To gain experience with the basic functionalities of GA_v2, we recommend the users to consult the technical details described in the additional files of GA_v1.19 [5] and also the Manual.pdf available in the docs folder. All of the scripts are available in the folder scripts and are open source as GA_v2. In order to make the most of GA_v2, we created four .mp4 videos with Kazam application (https://launchpad.net/kazam; accessed on 31 August 2022), which are available as Supplementary Materials (Video S1–S4).

Although the results of a JQ alignment may be reported from left to right in the GUI either in the red-blue or blue-red order (depending on the orientation of the insertion), we will further use the expression “blue-red” for both alternatives. The TGN represents the first blue nucleotide next to a red NT nucleotide at the genome–transposon junction border/juxtaposition, and it is highlighted in green. 

The nucleotide sequences uploaded in the genome database (GD) or in the transposon database (TD) of GA_v2 are indexed as reference sequences; therefore, any query sequence may be aligned against each of these references. Whenever one introduces in the GD a collection of contigs standing for a new sequenced genome, each of the reads/contigs is indexed as a reference sequence. Similarly, if the canonical release of a model genome (such as r6.47 of *D. melanogaster*) is deposited in the GD, each of the chromosome sequences is implicitly converted into an indexed reference sequence. To avoid confusions between the canonical reference assembly of a model genome and the genome assembly generated in a new sequencing project, we will further use the terms canonical reference genome (CRG) and alternative reference genome (ARG) to describe the workflows and performances of GA_v2. 

The loading time (see Video S2: Loading_Genomes.mp4) of a reference genome in the GD varies from a few seconds (for a bacterial genome) to a few minutes for a eukaryotic genome of moderate size (such as of *D. melanogaster*). Obviously, for a mammalian genome, the loading time could rise to even a few tens of minutes. We recommend using a computer equipped with at least 16 Gb of RAM, 500 Gb SSD and a processor able to work at around 3.5–4.00 GHz. It may be noticed that the mid-range laptops fulfill these requirements; the performance of GA_v2 is significantly increased when it is run on a more powerful computing machine. The mapping results we present herein are obtained by working with regular computers, equipped with Linux Mint or Ubuntu OS, 16–64 Gb of RAM, Intel or AMD processors of maximum 4.5 GHz, and no more than 1 Tb of SSD.

The time required for aligning and assembling the results depends on the computing power and also on the number of the queries (a query sequence or a list of many queries) run against the reference genome, the length of each query and the size of the genome. The best performances are obtained when the reference genome is interrogated with queries of tens to a few hundred/thousands of nucleotides. We do not recommend the usage of query sequences longer than 15,000 nucleotides.

A key issue is setting the parameter named Nucleus size (Settings/Parameters/Nucleus size). For query sequences longer than a few hundreds of nucleotides, a higher value than 10 (the minimal effective value) should be set for the Nucleus size in order to obtain the alignment results in a reasonable time-span. As an example, when the Nucleus size is set to 15–20, the user might obtain in just a few minutes a list of alignments for the interrogation of a moderate size reference genome with a 10,000 nucleotides query. An indication that the Nucleus size is used at values that are too low (10–15) for the length of the aligned queries is that the aligning process freezes at the stage assembling results shown in the status bar. In this case, the software should be restarted and a higher value of the Nucleus size may be tested. A drawback of GA_v2 that we aim to fix is that only one session of GA_v2 can be run at once. When two sessions of GA_v2 are simultaneously open, some RAM issues would occur and the computer must be restarted. 

Picking depth parameter is key when searching if more than one insertion of an NT occurs in a reference sequence, regardless of whether the reference is a long read, a contig, or an assembled chromosome. By default, the Picking depth parameter is set to the value of 1. Therefore, once a query sequence is covered by an alignment versus a subsequence of a particular reference sequence, no other similar alignment is reported, even if identical subsequences occur at different coordinates. If there are two instances of an NT insertion in a contig, only one of them will be reported when Picking depth is set to 1.

We further detail two recommended pipelines for mapping NTs, namely Workflow 1 (WF1) and Workflow 2 (WF2), designed to map insertions of hobo and P mobile elements harbored by the genome of *D. melanogaster* Horezu strain.

We chose to work with P-element and hobo NTs because they are both class II transposons and produce 8 bp length TSDs. Additionally, P elements spread relative recently in natural populations of *D. melanogaster* [12] but are completely absent in the reference genome. This aspect facilitates the identification of the P-element with GA_v2, as all the results obtained for a given ARG will be marked in blue-red and have a green TGN. Apart from the P-element, hobo is found in many genomic locations in the reference genome (~ 60 annotated copies). We included it in this study to exemplify the two types of output that GA_v2 offers when dealing with transposons existing in the reference: a blue alignment for those conserved and a blue-red output for those identified only in the analyzed ARG.

Each workflow makes use of specific GA_v2 packages and associated scripts. The value for the parameter Number of results impacts on results visualization. For WF1, we recommend a value around 1000 in order to find more positive results for NTs remnants. For WF2, the alignment score of a NT insertion with an almost intact TIR next to the green TGN is increased with 500 if the bonus option is activated. Therefore, a list of two results is usually enough to map an insertion of a DNA transposon. After finishing the WF1, the user must close the GA_v2 containing the ARG and only then start the WF2 with the GA_v2 package loaded with the CRG and the NTs reference sequences. 

The WF1 package contains only the ARG in the GD and the user scans it with a query sequence represented by a selected subsequence of it, in order to find contigs with insertions of the NT. In this stage, there is no need to have the NT reference sequence loaded in the TD. Herein, we present examples for finding and mapping of hobo and P-element insertions in a collection of contigs generated with Flye tool [13] starting from two sets of reads obtained by sequencing and re-sequencing of the Horezu strain. For each transposon, we used two query sequences, where the first one is represented by the first 60 nucleotides cropped from the 5’ end (Q5’) of the NT, and the second one contains the last 60 nucleotides from the 3’ (Q3’) end of the NT. The nucleotide length of these queries is an arbitrary one, but this interval of 60 nucleotides contains the TIRs of P-element (31 bp) or of hobo (12 bp), plus a subsequence that allows us to differentiate between the 5’ TIR and 3’ TIR of an NT when the insertions are in either I or II orientation.

The alignments that cover almost the entire query length and have a reasonable score (the user picks the threshold score; for a 60 nucleotides query, we recommend a score of at least 40) are visually checked to have the complete TIR. Then, they are exported as tabular data and are manipulated with specific scripts to export junction sequences required as queries in the WF2. A junction sequence is composed by either a Q5’ or a Q3’ plus flanking nucleotides extracted from the respective contig. The length of the flanking region is decided by the user and the size of the upstream flanking sequence of Q5’ may be different from the length of downstream flanking sequence of Q3’. Consecutive to our tests, we concluded that at least a few hundred or even thousands of nucleotides should be considered for extension if the NT is inserted in a repetitive sequence (such as in a different NT) or if the remnant is located quite close to an end of the ARG contig. The extraction script exports an ALERT string in the header of any contig sequence containing terminal remnants. 

Frequently, GA_v2 detects positive alignment hits for both Q5’ and Q3’, but for some NT remnants it finds only a Q5’ or a Q3’, since one TIR has been lost during evolution. For each insertion having both TIRs, the script extracts an upstream flanking sequence for Q5’ and a downstream sequence for Q3’, regardless of whether the NT is inserted in the relative orientation plus (orientation 1) or minus (orientation 2). As a result, a collection of junction sequences (JQ5’s and JQ3’s) is constructed. A particular issue is found when an NT remnant has only one TIR; in this case, the extraction procedure is identical, as above, for (let us say) the present Q5’. However, to extract the paired JQ3’, a different procedure joins three consecutive subsequences: the Q5’ one, a downstream subsequence with the same length as the respective NT, and 2000 (adjustable) additional downstream nucleotides. 

The recommended settings of the parameters for this step are Short, Nucleus size = 20–30 and Picking depth = 1.

In order to map hobo and P transposons in Horezu strain, we opted for collecting 3000 nucleotides long flanking sequences; therefore, the 5’ and 3’ JQ sequences had approximatively 3060 nucleotides. The JQ5’s and JQ3’s generated in WF1 were used as queries to map the insertions of the NT in the ARG, but versus CRG. The procedure requires a second GA_v2 package, containing only the CRG in the GD and the reference sequence of the NT in TD. We recommend activating the bonus option in this stage in order to prioritize the blue-red results in the list of exported alignments.

The collection of JQs generated by WF1 and used to map TIR-containing insertions of P-element and hobo in Horezu ARG versus r6.47 CRG in WF2 are provided in the Supplementary Materials (Files S1 and S2).

**Figure 5:**
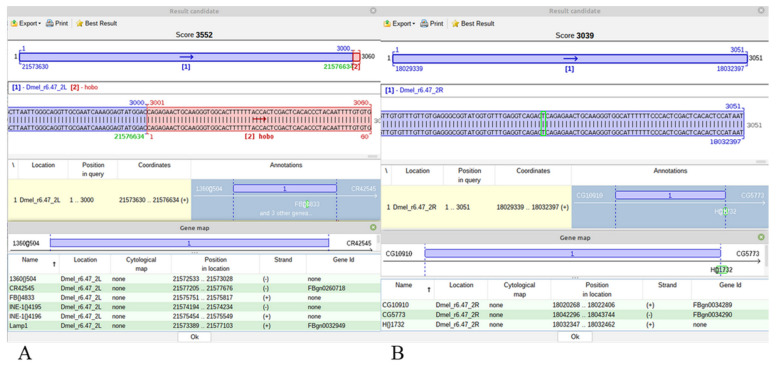
Mapping of two distinct hobo JQ5’s sequences. (**A**) Panel A depicts a Horezu specific insertion within *Lamp1* gene. (**B**) Panel B shows a hit in a reference mapped hobo TE, H{}1732. The genomic nucleotide adjacent to the first nucleotide of the 5’ TIR of hobo is manually highlighted with a green rectangle.

For a hobo insertion, a blue-red best scoring alignment is obtained only when it is specific for the Horezu strain, the ARG (Figure 5A). Alternatively, a similar/identic insertion may exist in the CRG but it may be either annotated or not in FlyBase. In both cases, the best alignments of JQ5’ or JQ3’ are completely blue best scoring results (Figure 5B). In these cases, no green TGN is to be highlighted, but the user can visualize the genomic region of insertion and may also easily compute the TGN coordinate, which is the first genomic nucleotide next to nucleotide 1 of Q5’ or to nucleotide 60 of Q3’.

The rationale of this output is that if a best blue scoring alignment covers the length of a JQ over a nucleotide interval of CRG, then GA_v2 does not compute any other shorter alignments in the same coordinate range of the query or reference sequences. Obviously, an alternative blue-red alignment with the same score is excluded in this case. 

Using the aforementioned strategy, we have successfully mapped 25 hobo insertions (Table 2). Fourteen hobo insertions are present only in Horezu genome and six genes are hit by insertions, namely *Lamp1*, *Nlg4*, *Mnr*, *CG42541*, *dnc* and *bru3*. The remaining 11 hobo insertions were found in both Horezu and *D. melanogaster* r6.47 reference genotypes; 2 of them hit *Stlk*, *fog* and *CG42346* genes. Out of these 11 insertions, only 6 are currently annotated in the reference genome. The other five hobo insertions hit four genes (*l(3)80Fg*, *Myo81F*, *Pzl* and *MFS17*) and represent new annotated insertions mapped by GA_v2. 

The mapping of the P-element is simpler because this NT is absent in the CRG of *D. melanogaster*; therefore, a JQ alignment is never a completely blue one. Since only the CRG fragment is covered by a blue sub-alignment of the JQ, the rest of the query is aligned versus the P-element reference sequence; therefore, a blue-red best scoring alignment is reported and the TGN is highlighted in green.

**Figure 6:**
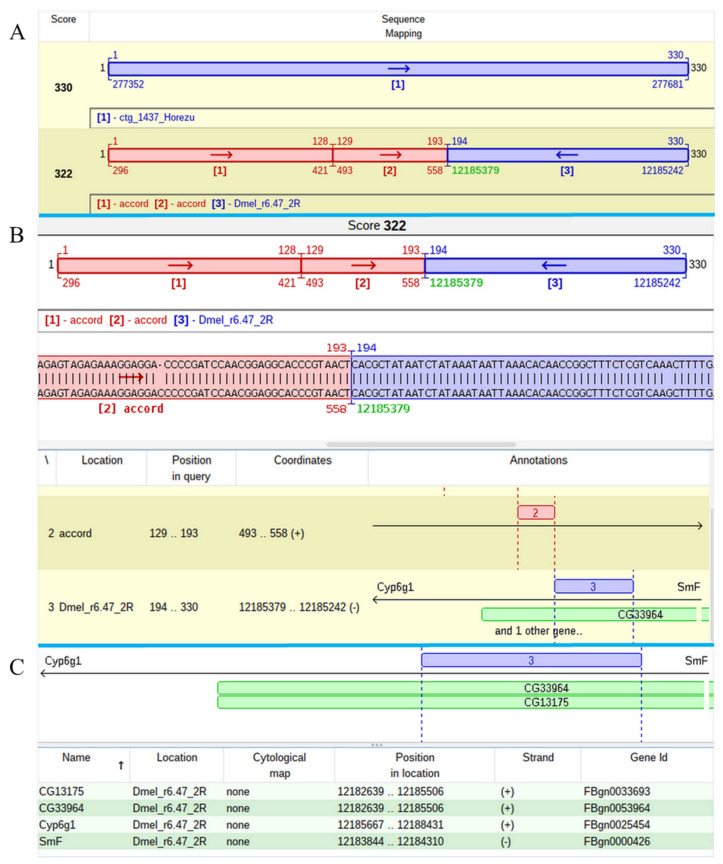
Mapping of an accord insertion in overlapped *CG13175* and *CG33964* genes of Horezu strain. (**A**) Panel A shows the mapping of the specific JQ in both Horezu 1437 contig and *D. melanogaster* reference genome. The green coordinate represents the TGN. (**B**) Panel B details the accord mapping, relative to the reference genome, while (**C**) panel C highlights the genomic context of this insertion by showing the hit genes, as well as the genes residing in close vicinity, *Cyp6g1* and *SmF*.

The mapping of one of two P-element insertions that was found in the contig 1437 of Horezu strain was a challenging task. The alignment of the respective JQ5 points to an insertion in the overlapped genes *CG13175* and *CG33964*, but the result of the JQ3 analysis indicates that this insertion is located in *Cyp6g1*, a gene located in the close proximity of *CG13175*/*CG33964*. The poor quality of the subalignments close to both TIRs of the P-element led us to manually annotate these regions and to find that they are actually fragments of the accord LTR retrotransposon. Basically, this P-element insertion hit an accord insertion located in its turn in the overlapped genes *CG13175*/*CG33964* (Figure 6). 

Interestingly, an accord insertion in *Cyp6g1* is reported for various natural populations of *D. melanogaster* and this insertional allele confers resistance to insecticides [14,15,16]. We presume that this insertion of accord in *CG13175*/*CG33964* is actually the insertional allele previously reported as being located at 291 bp upstream of the transcription start site of *Cyp6g1* [14,15,16]. The increase in the mapping resolution obtained with GA_v2 is consistent with the fact that the nucleotide coordinate corresponding to 291 bp upstream of the transcription start site of *Cyp6g1* is actually very close to the TGN 12185379, pertaining to *CG13175*/*CG33964* genes.

**Figure 7:**
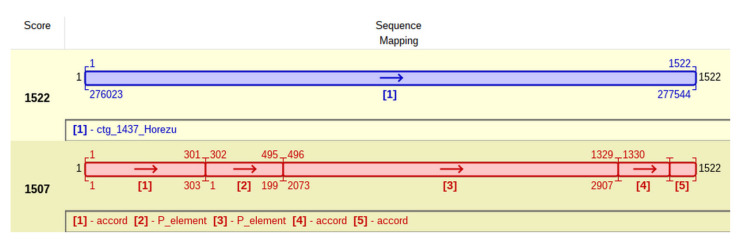
The specific JQ, collected from contig 1437 of Horezu strain (the blue alignment), is actually composed of accord ([1]—accord, [4]—accord and [5]—accord) and P ([2]—P_element and [3]—P_element) subsequences that allow the user to spot the internal deletions of each NT (the red-red alignment).

This particular insertion of P-element is located at coordinate 303 of accord (Figure 7) and generates a TSD with the approximate sequence GTCTAGAC. It is tempting to ask if this peculiar P-element insertion, located in an accord insertional allele, commonly found in many *D. melanogaster* natural populations, impedes on the adaptative resistance to insecticides of Horezu strain. 

Regarding the P-element, all of its insertions are found, as expected, only in the Horezu strain (Table 3) and affect four genes (*Ac13E*, *spri*, *shn* and *NFAT*).

In conclusion, this straightforward mapping strategy is true when the NT subsequence of a JQ is present in the ARG, but not in the CRG (as for the P-element), or when an NT is present in both the ARG and the CRG, but with instances of NT insertions present only in the ARG (the hobo case).

There is a distinction between mapping retrotransposons and DNA transposons, since DNA transposons are usually located in unique genomic regions or in low copy number sequences, such as genes, while retrotransposons are mainly located in repetitive sequences and often have repetitive sequence structure. Software, such as LoRTE (a tool also tested on *D. melanogaster* in the describing paper) [17] and RepeatMasker [18], only report the chromosome and the coordinates of each resolvable insertion, a task readily performed by GA_v2, but these tools do not offer detailed local genomic annotations. Instead, GA_v2 is able to map insertions based on junction sequences with the highest available resolution [5]; it reports detailed annotations in both GUI and in the tabular format; it works with any genome, regardless of its size; it works with any NTs sequence, regardless of its length; it may be run by using either fragments of an NT or with the whole reference sequence of an NT as a query; it is able to work both as a transposon tracker, performing the two workflows described in this paper, or as a standard aligner for nucleotide sequences, aligning certain sequences to one or more reference genomes (or the user can load a sequenced genome in the form of a contig list, in order to scan it with a query).

In contrast to the aforementioned mapping tools, GA_v2 is designed for the analysis of individual NTs of interest, not for a concurrent inquiry of the global NTs landscape of a genome. Although the user may load several genomes and transposons in GD and in TD, in both WF1 and WF2 a single transposon is processed, a mapping strategy, which, in our opinion, is less estimative and offers higher precision and more details about an NT of interest. 

Due to the structural complexity of the LTR elements, the extraction of JQs in WF1 must be done carefully, which implies a step of manual curation by the user. In the following example we describe both the identification and mapping of the copia retrotransposon, as well as the potential issues encountered in WF1 or WF2. The LTRs of copia are 276 nucleotides long, and in order to discriminate between LTR5’ and LTR3’, we used the terminal 600 nucleotides query sequences from both ends of copia. Some alignments almost completely cover the query length, but others stand only for an LTR; therefore, a manual inquiry should be performed in order to avoid ambiguities between the identical LTR5’ and LTR3’.

In order to check over the results of the mapping strategy performed with Q5’ and Q3’ derived from the copia LTRs, we repeated the procedure starting from a single query represented by a subsequence of 100 nucleotides extracted from the middle of the copia reference sequence. The specific JQs derived from this subsequence allowed us to remap copia in Horezu genome and the two sets of results coincide. In WF2, we achieved better results when Give bonus to insertion candidates was not activated, because the number of concurrent blue-red alignments was reduced.

Thus, GA_v2 found 14 copia insertions harbored by 13 contigs. Out of them, four were unresolvable, while ten were mapped at least at chromosome level (Table 4). Eight out of ten copia insertions are particular for the Horezu strain (blue-red alignments) and affect three genes (*Pzl*, *Dscam4* and *Pkc53E*). The remaining two insertions are present in both Horezu and the reference genome and map in Y chromosome (blue alignments). The apparent contradiction seen for contig 890, i.e., two distinct copia transposons mapping to different chromosomes, is most probably a consequence of genome missassembly. Comparatively, LoRTE only finds four copia insertions in the Horezu strain. This difference reveals a higher efficiency of GA_v2, at least for mapping this retrotransposon (Table 5).

GA_v2 is also able to perform a high-throughput processing of a retrotransposon for further mapping and polymorphisms analysis. Nevertheless, the user should be warned that the mapping of overrepresented NTs involves various issues, which become more complex as the genome of interest is larger and the particular retrotransposon to be mapped occurs in very high number copies, such as SINEs and LINEs in humans. These problems are intrinsic for other mapping software, as they rely on specific flanking sequences, regardless of whether these sequences are only TSDs or extended sequences. To map either DNA transposons or retrotransposons with high accuracy, GA_v2 requires unique flanking DNA sequences which border the insertion. The key step of extracting specific flanking sequencing is performed in WF1, but care should be taken when interpreting the alignments of the respective JQs, since retrotransposons are often inserted in highly repetitive regions, such as similar or different NTs. Therefore, many flanking sequences bordering retrotransposons insertions may align almost perfectly in several places in the genome, preventing the user to map some insertions from an ARG at unique locations in the CRG. These cases may sometimes be solved by extending the length of the extracted flanking sequences and a manual curation of the GA_v2 output file. By default, some insertions cannot be mapped, either because of inherent sequencing and assembly issues, or due to their occurrence inside very long repetitive genomic landscapes.

A particular case is when the first few high-scoring alignments in the list of results for a particular JQ have very similar scores (differences of the magnitude order of tens) and map at various genomic nucleotides coordinates pertaining to the same chromosome of the CRG. Obviously, such insertions can be mapped at chromosome resolution, but not at genomic nucleotide coordinate or gene resolution. 

To facilitate the interpretation of the colored results offered by GA_v2 in GUI, we further describe some technical aspects specific for the aligning and mapping strategy of GA_v2. 

The two databases of GA_v2 are versatile and one may decide which database should be used to upload reference sequences. The same reference sequence may be uploaded in any of the databases or in both of them, either to have a positive control for a target alignment, or to get rid of the genomic restrictions. In order to have two (or more) blue subalignments coalesced in the same final alignment, there are two rules to be fulfilled. First of all, two blue subalignments are forbidden to share overlapped genomic coordinates (genomic distance ≥ 0). Then, only if the first condition is fulfilled, the maximal genomic distance between two subalignments should not be bigger than a customizable value (set in the source code and requiring the compilation of a new GA_v2 package when changed), which by default is 200. Therefore, whenever the query sequence is a gene/NT affected by a deletion > 200 nucleotides, GA_v2 would report two different final results for each of the two genic fragments, which border the deletion. 

These two restrictions are not functional when GA_v2 composes blue-red or red-red alignments to allow the detection of NTs insertions, NTs self-insertions and large internal deletions affecting some transposon remnants. Care should be taken when contigs are loaded in the NT database, since they would be considered as transposons and there are no restriction rules. This may be advantageous when the user wants to check how many remnants of an NT are present in a long contig, as they would be composed in the same final alignment. On the other hand, if an NT remnant is affected by a big internal deletion, then the two partial alignments are presented as two discrete subalignments relative to the length of the NT reference sequence. It is always instructive to analyze the partial results used to compose the final results; in such a case, one may notice that the two transposon fragments are combined in the same alignment, regardless of whether they share overlapped nucleotide coordinates of the NT reference sequence. 

Summarizing, GA_v2 is able to identify NTs insertions in long reads, i.e., generated with nanopore technologies, as well as in contigs generated by the assembly of reads obtained with previous generations of sequencing technologies. To scan for transposon insertions, the user must load the reads or the ARG contigs sequences in the GD and then use sets of Q5’ or the Q3’ sequences as queries. If a complete NT canonical sequence is used instead to scan for insertions, the WF1 should be adapted by the user in order to obtain equivalent JQs. Most of the existing tools that identify transposable elements in sequenced genomes involve dependencies that are usually of two types: databases (more or less available and updated to the latest genome version) and applications/packages (which can undergo changes or may become obsolete over time, leading to problems with the tool’s functionality).

An obvious advantage of GA_v2 is a friendly graphical user interface (GUI), which allows the user to visualize and to interpret the results of aligning and mapping, along with various genome annotation features. Most mapping programs do not have a graphical interface, hence raw alignment results are difficult to obtain and interpret. An example is LoRTE software, dedicated to the analysis of long read sequences and able to identify the presence/absence of NTs in a sequenced genome, comparative to a reference genome [17]. LoRTE is written in Python 2.7 and depends on a recent version of the ncbi-blast+ package and BioPython. To run the program, five input files are needed: the reference genome, a .fasta file with reads, a list of annotated NTs (difficult to obtain for some species or for newer versions of genomes), a list of consensus NTs and a list of parameters that the user modifies according to their choice. The output does not include the actual alignments, but rather lists present/absent/new inserted NTs, with or without sequence polymorphisms. LoRTE indicates the chromosome and the coordinates where the NTs are located, but it does not show annotations of the local genomic landscape (the presence of genes or other NTs, TSDs, etc.). A drawback of LoRTE is that it cannot be run without the lists of consensus NTs or NTs annotations specific for the reference genome.

LoRTE does not use junction sequences, instead using only flanking sequences for the NTs and checking them on the CRG with MEGABLAST [17]. On the contrast, GA_v2 uses JQs, which are a hallmark of our tool, which improves the resolution of mapping insertions with the highest resolution reported so far [5], mainly when partial overlapping occurs between the IRs or LTRs and the proximal ends of the flanking sequences. Although GA_v2 is able to detect polymorphisms, such as new insertions/deletions of an NT in a population versus the reference genome, it is mainly focused on a comparative and very accurate mapping and annotation of each insertion of an NT of interest. As a result, GA_v2 offers mapping at chromosomal and/or genic level, and it does not compute the total NTs content in numbers and percentages.

Our TIR-dependent mapping strategy identified the same conserved hobo remnants in CRG r6.47 that were identified by LoRTE in an older reference genome (r5), for which the file with TE annotations is available. Apart from the hobo remnants that have one of the TIRs and which were identified with both GA and LoRTE, the latter tool also detected a number of remnants without TIRs, but which have internal sequences specific to the hobo transposon. The mapping strategy of GA_v2 described herein is customizable and may be adapted to search for NTs remnants, which have no TIRs. To initiate the WF1, various internal subsequences of the NT reference sequence can be selected and used as alternative queries to search for remnants that have been missed by TIR-dependent mapping strategy. 

In summary, GA_v2 outperforms LoRTE when single transposons are mapped, regardless of their mechanism of transposition (Table 5). 

Another NTs mapping tool without a GUI is the transposon insertion finder (TIF), which was designed to identify insertions by using as queries the terminal sequences of NTs [19]. A consistent limitation of this mapping tool is the exclusive requirement of TIF for short reads generated by Illumina, which are grouped by the presence of a specific TSD sequence.

When GA_v2 is compared with RepeatMasker, a program generally used to evaluate the content of transposons in sequenced genomes [18], one can notice that the work strategies and the expected results are different. Unlike RepeatMasker, GA_v2 allows a detailed analysis of a specific NT of interest just by using an NT subsequence as a query to be run versus a database containing reads or contigs set as reference sequences. Both tools allow the user to find out the number of copies of an NT of interest, but RepeatMasker is able to mask the repetitive sequences in the analyzed genome and to calculate the proportion of total transposable elements in the genome. An advantage of RepeatMasker is the online version with GUI, but with a size limitation of 100 kb for the contigs scanned for insertions. To obtain accurate alignment and mapping results, GA_v2 relies on its own alignment heuristic described in detail elsewhere [5]. Conversely, RepeatMasker can be run via an implementation of the Smith–Waterman algorithm or via BLAST aligner, an alternative that conducts to a decrease in the tool’s sensitivity. Even though RepeatMasker is one of the most used programs in the analysis of repetitive sequences, it also has some limitations concerning the accuracy of the identification of NTs residues [20].

We verified if the EXTRACTION.sh script used in WF1 is functional on the genomes of different species downloaded from other database than FlyBase. For this purpose, we tested the script on the genome of *Caenorhabditis elegans* from Ensembl release 107 [21] and concluded that the collection of the JQs was generated as expected. Therefore, the mapping strategy of GA_v2 is not limited to *D. melanogaster* or to the FlyBase format. Unfortunately, to our knowledge, there are no annotations for NTs available in Ensembl; therefore, GA_v2 does not show the NTs local landscape for the mapped insertions.

## 3. Materials and Methods

GA_v2 is open-source software compatible with Linux, Mac OS and Windows and is available at https://github.com/genomeartist/genomeartist and at www.genomeartist.ro (accessed on 31 August 2022).

Implementation of the new functions in GA_v2 (Genome ARTIST v2.0.2—DOI: 10.5281/zenodo.4139251) has been performed in the *Java* programming language. Using *Apache Ant* compiler and OpenJDK 8, GA_v2 can be compiled on Linux, Mac OS and Windows. 

The individual files containing the computed alignments are saved as files.ga in resources/outputs folder, and they can be visualized by loading them even in an empty GA_v2 package, sparing the time required to run the respective alignments.

The new scripts of GA_v2 are EXTRACTION.sh, UPSTREAM_EXTRACTION.sh, DOWNSTREAM_EXTRACTION.sh, UPSTREAM_TE+GENOMIC_EXTRACTION.sh and DOWN-STREAM_TE+GENOMIC_EXTRACTION.sh, and all of them were written in *bash*, using Linux Mint and Ubuntu environments. The scripts are deposited in the scripts/Junction_Queries_Extraction directory and may be run in Linux or Mac OS in order to generate the junction sequences, which can be subsequently used for mapping on Windows OS also. Alternatively, the scripts are effective on the application Windows Subsystem for Linux, available in the software store of Windows 10. The source code of the software, compilation instructions, related scripts, as well as the manual of the tool can be obtained from the genomeartist repository available at the address https://github.com/genomeartist/genomeartist (accessed on 31 August 2022).

Detailed instructions for general GA_v1.19 installation and running (also available for GA_v2) are described in a former paper [5], in the manual available in Documents folder and on the dedicated website (http://www.genomeartist.ro/, accessed on 31 August 2022). To familiarize the user with the workflows executed by GA_v2 to map NTs in ARG contigs, as well as against CRG, we conceived 4 short videos recorded with Kazam (Supplementary Materials: Video S1–S4).

In order to load in GA_v2 a collection of ARG contigs obtained after the assembly step, the contigs should be converted from the standard FASTA into RAW format compatible with GA_v2. For this step, the user needs to run the ConvertFASTAtoRAW.sh script on a multi-FASTA file, which contains the sequences of the contigs. This script is available in the scripts/ARG_Conversion directory. To be correctly interpreted by GA_v2, the header structure should begin with “>contig_number”.

Starting from genomic coordinates exported in tabular format consecutive to queries mapping, these scripts extract the JQs. We mention that, in order to obtain a table that can be used by the scripts to generate JQs, the header of any Q5’ or Q3’ should end with “TIR5” or “TIR3” (header example: >P_element_TIR5). To obtain the junction sequences, only the EXTRACTION.sh script must be run, but all five of them should be marked as executable. The two tables associated with each Q5’ and Q3’ of an NT are exported by GA_v2 and should be present in the working directory (we recommend scripts/Junction_Queries_Extraction) along with the ARG contigs in a multi-FASTA file format.

In our study, when mapping hobo and P-element the GA_v2 running parameters for WF1 were as follows: Type of interval extension = Short, Picking depth = 1, Nucleus size = 20, Number of results = 1000, Give bonus to insertion candidates is not checked; all the other variables had default values. The running parameters for WF2 were as follows: Type of interval extension = Short, Picking depth = 1, Nucleus size = 30, Number of results = 2, Give bonus to insertion candidates is checked; all the other variables had default values. When mapping copia, the GA_v2 running parameters for WF1 were as follows: Type of interval extension = Short, Picking depth = 10,000, Nucleus size = 20, Number of results = 1000, Give bonus to insertion candidates is not checked; all the other variables had default values. The running parameters for WF2 were as follows: Type of interval extension = Short, Picking depth = 5, Nucleus size = 30, Number of results = 20, Give bonus to insertion candidates is not checked; all the other variables had default values.

The Q10 filtered reads corresponding to Horezu strain sequencing and re-sequencing are associated with BioProject/NCBI—PRJN629549. The actual data can be accessed by accessing the NCBI/Sequence Read Archive: SRX8215201. From here, for Horezu sequencing data (Q10 filtered nanopore reads), access Run: SRR11654246; for Horezu re-sequencing data (Q10 filtered nanopore reads), access SRA study: SRP259353.

The draft Horezu genome assembly was obtained with Flye 2.8.3 loaded with two collections of Q10 filtered reads that are associated with the aforementioned BioProject. This Whole Genome Shotgun project has been deposited at DDBJ/ENA/GenBank under the accession JANZWZ000000000. The version described in this paper is version JANZWZ010000000.

## 4. Conclusions

The GA_v2 is a reliable support tool for at least class II transposons annotation. In addition to all previous performances of GA_1.19, the new version is also able to map TGN with the highest precision reported so far in the literature. The ability to map self-insertions and to detect nested insertions are also a unique asset of our tool [5]. GA_v2 was improved to facilitate the high-throughput analysis of JQs data, regardless of whether they were generated by insertional mutagenesis projects (mapping of artificial transposons) or by genome sequencing (mapping of NTs).

Compared to the other NTs mapping tools, GA_v2 has consistent strengths. GA_v2 may be uploaded with a genome containing both genes and NTs annotations in GD (if they are available, as for *D. melanogaster* in FlyBase); therefore, any new insertion (polymorphism) from a natural population may be analyzed in the context of the local NTs landscape. GA_v2 not only confirms if a specific NT insertion is present in the ARG comparative to CRG but also allows the user to work with any personalized list of NTs or with just one or a few NTs of interest. GA_v2 is an autonomous NTs mapper since it does not depend on the implementation of other aligners, such as BLAST or BLAT and relies on its specific scripts written in *bash*. The only dependencies of GA_v2 are: the contigs of the ARG, the appropriate CRG and the reference sequences of the NTs of interest. GA_v2 may be used with any kind of sequences defined as references, regardless of whether they are amplicons, long sequencing reads, contigs or canonical chromosomes and transposons. The GUI is friendly and informative and allows the regular user to visually interpret the alignments data and also to export a customized name.csv table with a variety of mapping and annotation data, which may be further processed with other bioinformatics tools or in *bash*.

The quality of P-element and hobo mapping on contigs derived from long reads generated by nanopore sequencing of *D. melanogaster* strain from Horezu, Romania, reveals that GA_v2 is a reliable tool for tracking and annotation of NTs in genomes of interest.

Various tools are employed for NTs mapping in either sequencing reads or in assembled genomes. To our knowledge, each of them has some drawbacks, so there is a permanent bioinformatics work aimed to develop new tools or to optimize the integration of some of them in annotation pipelines [22]. The benchmarking of the NTs mapping procedure is a continuous challenge for the scientific community [23]. In this context, we consider that one aspect of this demanding endeavor would be to even consider the offline tools, such as GA_v2, for comparative tests of performances, either when standing alone or when incorporated in pipelines dedicated for NTs mapping and annotation. The precision of GA_v2, along with its ability to map self-insertions and to generate consensus sequences for sets of TSDs or other flanking sequences, recommend it to be considered along other NTs mapping tools when annotation benchmarking tests are run.

## Figures and Tables

**Table 1:** The new features of GA_v2, compared with GA_v1.19.

Features	GA_v1.19	GA_v2
Cross-platform compatibility	No	Yes
Instruction set architecture	x32	x64
Export tabular data	No	Yes
Extract flanking sequences	No	Yes
Export TSD sequences	No	Yes
Export consensus sequence	No	Yes
Scripts for NTs mapping	No	Yes
Browse button to load queries	No	Yes
Automatically name queries	No	Yes
Highlighted TGN	No	Yes

**Table 2:** Mapping of hobo NT in Horezu strain of *D. melanogaster* relative to the reference genome (r6.47).

Hobo			
Flye_Q10_Contigs	Insertions Present in *D. melanogaster* r6.47	Insertions Specific for *D. melanogaster* Horezu Strain	Hit Genes
232	-	21576627, 2L	*Lamp1*
439, 1409	H{}4523, 3R	-	-
592	H{}4006, 3L	-	-
595	unannotated hobo, 24774032, 3L	-	-
625	-	14998026, 2R	-
721	-	17551987, 2L	-
755	H{}4815, 3R	-	-
893	unannotated hobo, 25850090, 3L	-	*l(3)80Fg*
933	H{}3283, 2R	-	*Stlk*
1094	H{}2456, X	-	*fog*; *CG42346*
1345	unannotated hobo, 943127, 3R	-	*Myo81F*
1426	-	12963046, 2L	-
1492	unannotated hobo, 2628131, 3R	-	*Pzl*
1510	-	12300267, 3L	-
1539	unannotated hobo, 2495728, 2R	-	*MFS17*
1623	-	20235442, 3R	*Nlg4*
1957	H{}1732, 2R	-	-
2061	-	14998019, 2R	-
2062	-	14999459, 2R	-
2140	-	21225504, X	*Mnr*
2202	-	970719, X	-
2226	-	6026436, X	-
2260	-	3925353, X	*CG42541*
2439	-	3306632, X	*dnc*
2447	-	13627239, 3L	*bru3*

**Table 3:** Mapping of P-element in Horezu strain of *D. melanogaster* relative to the reference genome (r6.47).

P-element			
Flye_Q10_Contigs	Insertions Present in *D. melanogaster* r6.47	Insertions Specific for *D. melanogaster* Horezu Strain	Hit Genes
724	-	29765640, 3R	-
809	-	21832841, X	-
1068	-	2242343, 2L	-
1188	-	15552358, X	*Ac13E*
1191	-	3063077, 2R	-
1280	-	10547688, X	*spri*
1437	P/accord complex insertion in *CG13175* and *CG33964* (mapping details in the text)		
1535	-	11199636, 2R	*shn*
1646	-	19180624, 3R	-
2153	-	13641922, X	*NFAT*

**Table 4:** Mapping of copia element in Horezu strain of *D. melanogaster* relative to the reference genome (r6.47).

Copia			
Flye_Q10_Contigs	Insertions Present in *D. melanogaster* r6.47	Insertions Specific for *D. melanogaster* Horezu Strain	Hit Genes
529	-	3006576, 3R	*Pzl*
688	-	3642616, 3R	-
890	-	25188852, 3L	-
890	unannotated copia, 261724, Y	-	-
1166	-	unresolvable locus, rDNA	
1169	-	unresolvable locus, Y	-
1414	-	6323794, 2R	-
1620	unannotated copia, 1979889, Y	-	-
1972	-	8242829, 3L	*Dscam4*
2075	-	16928722, 2R	*Pkc53E*

**Table 5:** Comparative performances of GA_v2 and LoRTE when P-element, hobo and copia NTs are mapped in the Horezu strain of *D. melanogaster*. The term new insertions indicates the number of insertions particular to Horezu, while conserved insertions stands for the number of insertions shared by Horezu and the reference genome (r6.47).

NTs	GA_v2	LoRTE
P-element	10 new insertions	NA
hobo	11 conserved insertions	25 conserved insertions (11 ambiguous)
	14 new insertions	0
copia	2 conserved insertions (1 ambiguous)	4 conserved insertions (3 ambiguous)
	8 new insertions	0
**Mapping resolution**		
**Chromosomal level**	Yes	Yes
**Gene level**	Yes	NA

## Data Availability

Horezu strain sequencing project—NCBI/Sequence Read Archive: SRX8215201, Horezu sequencing—Run: SRR11654246, Horezu re-sequencing—SRA study: SRP259353.

## Supplementary Materials

The following supporting information can be downloaded at: https://www.mdpi.com/article/10.3390/ijms232012686/s1, File S1: File_S1_JQ_hobo_Horezu.fasta, File S2: File_S2_JQ_P_Horezu.fasta; Video S1: Video_S1_Running_GA.mp4, Video S2: Video_S2_Loading_Genomes.mp4, Video S3: Video_S3_GA_WF1.mp4, Video S4: Video_S4_GA_WF2.mp4; SupplementaryMaterials_Video_S1–4_legends.pdf.

## Draft Horezu Genome Assembly

DDBJ/ENA/GenBank under the accession JANZWZ000000000.

## References

[1] Feschotte C., Pritham E.J. (2007). DNA transposons and the evolution of eukaryotic genomes. Annu. Rev. Genet..

[2] Nelson M.G., Linheiro R.S., Bergman C.M. (2017). McClintock: An Integrated Pipeline for Detecting Transposable Element Insertions in Whole-Genome Shotgun Sequencing Data. G3: Genes|Genomes|Genetics.

[3] Bergman C.M., Quesneville H. (2007). Discovering and detecting transposable elements in genome sequences. Briefiengs Bioinform..

[4] Vendrell-Mir P., Barteri F., Merenciano M., Gonzalez J., Casacuberta J.M., Castanera R. (2019). A benchmark of transposon insertion detection tools using real data. Mob. DNA.

[5] Ecovoiu A.A., Ghionoiu I.C., Ciuca A.M., Ratiu A.C. (2016). Genome ARTIST: A robust, high-accuracy aligner tool for mapping transposon insertions and self-insertions. Mob. DNA.

[6] Gramates L.S., Agapite J., Attrill H., Calvi B.R., Crosby M.A., Dos Santos G., Goodman J.L., Goutte-Gattat D., Jenkins V.K., Kaufman T. (2022). FlyBase: A guided tour of highlighted features. Genetics.

[7] Crooks G.E., Hon G., Chandonia J.M., Brenner S.E. (2004). WebLogo: A sequence logo generator. Genome Res..

[8] Streck R.D., Macgaffey J.E., Beckendorf S.K. (1986). The structure of hobo transposable elements and their insertion sites. EMBO J..

[9] Linheiro R.S., Bergman C.M. (2008). Testing the palindromic target site model for DNA transposon insertion using the Drosophila melanogaster P-element. Nucleic Acids Res..

[10] Liao G.C., Rehm E.J., Rubin G.M. (2000). Insertion site preferences of the P transposable element in Drosophila melanogaster. Proc. Natl. Acad. Sci. USA.

[11] Linheiro R.S., Bergman C.M. (2012). Whole genome resequencing reveals natural target site preferences of transposable elements in Drosophila melanogaster. PLoS ONE.

[12] Kelleher E.S. (2016). Reexamining the P-Element Invasion of Drosophila melanogaster Through the Lens of piRNA Silencing. Genetics.

[13] Kolmogorov M., Yuan J., Lin Y., Pevzner P. (2019). Assembly of Long Error-Prone Reads Using Repeat Graphs. Nat. Biotechnol..

[14] Daborn P.J., Yen J.L., Bogwitz M.R., Le Goff G., Feil E., Jeffers S., Tijet N., Perry T., Heckel D., Batterham P. (2002). A single p450 allele associated with insecticide resistance in Drosophila. Science.

[15] Catania F., Kauer M.O., Daborn P.J., Yen J.L., Ffrench-Constant R.H., Schlotterer C. (2004). World-wide survey of an Accord insertion and its association with DDT resistance in Drosophila melanogaster. Mol. Ecol..

[16] Chung H., Bogwitz M.R., McCart C., Andrianopoulos A., Ffrench-Constant R.H., Batterham P., Daborn P.J. (2007). Cis-regulatory elements in the Accord retrotransposon result in tissue-specific expression of the Drosophila melanogaster insecticide resistance gene Cyp6g1. Genetics.

[17] Disdero E., Filee J. (2017). LoRTE: Detecting transposon-induced genomic variants using low coverage PacBio long read sequences. Mob. DNA.

[18] Smit A.F.A., Hubley R., Green P. RepeatMasker Open-3.0. 1996–2010. http://www.repeatmasker.org.

[19] Nakagome M., Solovieva E., Takahashi A., Yasue H., Hirochika H., Miyao A. (2014). Transposon Insertion Finder (TIF): A novel program for detection of de novo transpositions of transposable elements. BMC Bioinformatics.

[20] Arensburger P., Piegu B., Bigot Y. (2016). The future of transposable element annotation and their classification in the light of functional genomics—What we can learn from the fables of Jean de la Fontaine?. Mob. Genet. Elements.

[21] Cunningham F., Allen J.E., Allen J., Alvarez-Jarreta J., Amode M.R., Armean I.M., Austine-Orimoloye O., Azov A.G., Barnes I., Bennett R. (2022). Ensembl 2022. Nucleic Acids Res..

[22] Ou S., Su W., Liao Y., Chougule K., Agda J.R.A., Hellinga A.J., Lugo C.S.B., Elliott T.A., Ware D., Peterson T. (2019). Benchmarking transposable element annotation methods for creation of a streamlined, comprehensive pipeline. Genome Biol..

[23] Hoen D.R., Hickey G., Bourque G., Casacuberta J., Cordaux R., Feschotte C., Fiston-Lavier A.S., Hua-Van A., Hubley R., Kapusta A. (2015). A call for benchmarking transposable element annotation methods. Mob. DNA.

