# Supplementary material for: Genome ARTIST_v2—An Autonomous Bioinformatics Tool for Annotation of Natural Transposons in Sequenced Genomes
Source: Int J Mol Sci. 2022 Oct 21;23(20):12686. doi: 10.3390/ijms232012686 (PMC9604107; doi:10.3390/ijms232012686)
Supplement: Supplementary file 1 [file ijms-23-12686-s001.zip › SupplementaryMaterials_Video_S1-4_legends.pdf]

Videos S1-S4 - details

Video_S1_Running_GA.mp4 – The S1 video presents a concise tutorial describing the succession of steps required to run GA_v2. It includes (i) one of the sources for GA_v2 download; (ii) the instructions for starting GA_v2; (iii) how to make the conversion of FASTA files into GA_v2-specific raw files; (iv) a brief presentation of GA_v2's graphic interface; (v) how to load an ARG in GD.

Video_S2_Loading_Genomes.mp4 – The S2 video shows how to download various drosophilid genomes from FlyBase/Ensembl along with genes/TEs annotations. It also details the use of specific scripts for processing the downloaded data and how to add the resulting files into the Genome files of GA_v2.

Video_S3_GA_WF1.mp4 – The S3 video displays an example of how to implement the WF1. The video starts with the scan of an ARG with a query sequence represented by a subsequence of a P-element, as a prerequisite to find contigs harboring NTs insertions. Subsequently, the alignments coordinates are retrieved and used for extraction of JQs.

Video_S4_GA_WF2.mp4 – The S4 video describes a case of a P-element mapping, where the JQs generated by WF1 are aligned to the *Drosophila melanogaster* reference genome during WF2. This video illustrates how to visualize the genomic context in which the NT is inserted, how to export the results in a tabular form and how to upload GA_v2 alignments in GUI.
